# Global Vaccine Action Plan lessons learned III: Monitoring and evaluation/accountability framework

**DOI:** 10.1016/j.vaccine.2020.05.028

**Published:** 2020-07-14

**Authors:** Thomas Cherian, Angela Hwang, Carsten Mantel, Chantal Veira, Stefano Malvolti, Noni MacDonald, Christoph Steffen, Ian Jones, Alan Hinman

**Affiliations:** a MMGH Consulting GmbH, Kuerbergstrasse 1, 8049 Zurich, Switzerland; b Angela Hwang Consulting, P.O. Box 6601, Albany, CA 94706, USA; c Task Force for Global Health, 330 West Ponce de Leon Ave., Decatur, GA 30030, USA; d Dalhousie University, IWK Health Centre, Halifax, Canada; e World Health Organization, 20 Avenue Appia, 1211 Geneva 27, Switzerland; f Jinja Publishing Ltd, Bishop’s Stortford, United Kingdom

**Keywords:** Global Vaccine Action Plan, Immunization, Monitoring and evaluation, CSOs, Civil Society Organizations, DoV, Decade of Vaccines, GVAP, Global Vaccine Action Plan 2011–2020, IA2030, Immunization Agenda 2030, M&E/A, Monitoring and Evaluation/Accountability, RITAG, Regional Immunization Technical Advisory Group, R&D, Research and Development, SAGE, Strategic Advisory Group of Experts on Immunization, SO, Strategic Objective, UNICEF, United Nations Children’s Fund, WG, Working group, WHA, World Health Assembly, WHO, World Health Organization

## Abstract

**Introduction:**

The Monitoring & Evaluation/Accountability (M&E/A) framework of the Global Vaccine Action Plan (GVAP) was used to report progress annually to the World Health Assembly (WHA).

**Methods:**

Stakeholder feedback was obtained through five reviews consisting of surveys and semi-structured interviews conducted from 2017 to 2019. Participants consisted of individuals involved in the development and implementation of GVAP or its M&E/A process, national immunization managers, academics, representatives of non-governmental organizations, and civil society organizations.

**Results:**

The feedback was mixed and contradictory for some components, though most participants reported that the M&E/A process was a highlight of GVAP and a step in the right direction. Several of the goals and targets were considered aspirational and unrealistic for many countries. There were mixed responses on whether it promoted accountability, especially at the country level.

**Discussion:**

The mixed and contradictory views on the M&E/A processes and its impact suggested a failure of communication about its scope and intent. Though the process, especially the annual reporting to the WHA, kept immunization high on the global agenda, it failed to fully meet the expectations in promoting accountability. Engaging with countries to capture the local context in setting global goals and targets and promoting local M&E/A processes will be important to achieve accountability in the next decade.

## Highlights

• The M&E/A framework was a critically important element of GVAP.
• It was a cyclical process of monitoring, review, and recommendations for action.
• Though a step in the right direction, it failed to hold all stakeholders accountable.
• Several GVAP goals were aspirational and unrealistic for many countries.

## 1 Introduction

The Global Vaccine Action Plan 2011–2020 (GVAP) was intended to catalyse a global drive to minimize the burden of vaccine-preventable diseases in every country. In contrast to previous global immunization strategies, the GVAP was accompanied by a Monitoring & Evaluation/Accountability (M&E/A) framework [1], and an independent process to assess progress and report annually to the World Health Assembly (WHA) as called for in a resolution adopted in 2012 [2]. The M&E/A framework and process, which are described in more detail in an accompanying article in this issue of the journal [3], were seen by many as a critically important element of GVAP.

The monitoring process focused mainly on the indicators for each of the GVAP goals and Strategic Objectives (SOs). The list of indicators for each of the GVAP goals along with the targets as originally established are shown in Table 1a, Table 1b.

**Table 1a:** GVAP goals, indicators, and targets (as established in 2013).

Goal	Indicator	Target
1 Achieve a world free of poliomyelitis	1.1 Interruption of wild poliovirus transmission globally	2014
	1.2 Certification of poliomyelitis eradication	2018
2 Meet global and regional elimination targets	2.1 Neonatal tetanus elimination	2015
	2.2 Measles elimination	2015: 4 WHO regions 2020: 5 WHO regions
	2.3 Rubella/congenital rubella syndrome elimination	2015: 2 WHO regions 2020: 5 WHO regions
3 Meet vaccination coverage targets in every region, country and community	3.1 Reach 90% national coverage and 80% in every district or equivalent administrative unit with three doses of diphtheria-tetanus-pertussis containing vaccines	2015: all Member States
	3.2. Reach 90% national coverage and 80% in every district or equivalent administrative unit for all vaccines in national programmes, unless otherwise recommended	2020: all Member States
4 Develop and introduce new and improved vaccines and technologies	4.1. Licensure and launch of vaccine or vaccines against one or more major currently non-vaccine preventable diseases	2020: one or more
	4.2. Licensure and launch of at least one platform delivery technology	2020: one or more
	4.3. Number of low-income and middle-income countries (LMICs) that have introduced one or more new or underutilized vaccines	2015: at least 90 2020: all LMICs
5 Exceed the millennium development goal 4 target for reducing child mortality	Under 5 mortality rate per 1000 live births	2015: 2/3 reduction compared to 1990 2020: exceed 2015 target

**Table 1b:** Strategic objective indicators and targets (as established in 2013).

**Strategic objective**	**Indicator**	**Target**
1 All countries commit to immunization as a priority	1.1. Domestic expenditures for immunization per person targeted	Increasing trend
	1.2. Presence of an independent technical advisory group that meets defined criteria	Functional groups in all countries
2 Individuals and communities understand the value of vaccines and demand immunization both as a right and a responsibility	2.1. Percentage of countries that have assessed (or measured) the level of confidence in vaccination at subnational level**	Increasing trend
	2.2. Percentage of un- and under-vaccinated in whom lack of confidence was a factor that influenced their decision**	Decreasing trend
3 The benefits of immunization are equitably extended to all people	3.1. Percentage of districts with 80% or greater coverage with three doses of diphtheria-tetanus-pertussis containing vaccine	2020: all districts in all countries
	3.2. Reduction in coverage gaps between wealth quintiles and other appropriate equity indicator(s)	Increasing trend in equity
4 Strong immunization systems are an integral part of a well-functioning health system	4.1. Dropout rate between first dose (DTP1) and third dose (DTP3) of diphtheria-tetanus-pertussis-containing vaccines	Decreasing trend
	4.2. Sustained coverage of diptheria-tetanus-pertussis-containing vaccines 90% or greater for three or more years	2020: all countries
	4.3. Immunization coverage data assessed as high quality by WHO and UNICEF	2020: all countries
	4.4. Number of countries with case-based surveillance for vaccine-preventable diseases	2015: all countriesfor polio and measles 2020: 75% of LMICs for sentinel site surveillance
5 Immunization programmes have sustainable access to predictable funding, quality supply and innovative technologies	5.1. Percentage of doses of vaccine used worldwide that are of assured quality	2020: 100% of all vaccine doses
6 Country, regional and global research and development innovations maximize the benefits of immunization	6.1. Progress towards development of HIV, TB, and malaria vaccines	Proof of concept for a vaccine with ≥ 75% efficacy
	6.2. Progress towards a universal influenza vaccine (protecting against drift and shift variants)	At least one vaccine licensed
	6.3. Progress towards institutional and technical capacity carry out vaccine clinical trials	Every region with solid base
	6.4. Number of vaccines that have either been re-licensed or licensed for use in a controlled-temperature chain at temperatures above the traditional 2–8 °C range	Increasing number
	6.5. Number of vaccine delivery technologies (devices and equipment) that have received WHO prequalification against the 2010 baseline	Increasing number

** Provisional indicator to be finalized based on outcomes of pilot assessment in selected regions.

The World Health Organization (WHO) Strategic Advisory Group of Experts (SAGE) for immunization established the Decade of Vaccines Working Group (SAGE DoV WG) to conduct the annual assessment. The SAGE DoV WG prepared annual reports featuring progress summaries and recommendations for corrective actions that were reviewed and revised by SAGE and submitted to the WHA through the WHO Executive Board, where they were discussed as a substantive agenda item annually through 2018.

The Gavi civil society organization (CSO) constituency was used as a platform to obtain the annual reports from CSOs on their engagement in supporting immunization programmes at national and subnational levels. Other important actors were also invited to submit summaries of their organization’s contribution toward attaining GVAP goals.

In the latter half of the decade, as a result of the approval of Regional Vaccine Action Plans, M&E/A processes were established in all WHO regions, with the independent review being conducted by the Regional Immunization Technical Advisory Groups (RITAGs). The reports were presented to the respective Regional Committees, though not necessarily on an annual basis or as substantive agenda items. Information on country level monitoring processes is not available.

At the global level, WHO in partnership with other global immunization stakeholders convened meetings with country health delegations during the WHA to discuss progress and to advocate for action in response to SAGE recommendations. Beyond this, at the global level, there were no mechanisms to implement accountability specifically for GVAP. In the latter half of the decade, monitoring by the Regional Immunization Technical Advisory Groups (RITAGs) resulted in an accountability process at the regional levels.

Since it was an innovative component of GVAP, the M&E/A framework and process and its impact were specifically assessed as part of the GVAP evaluation.

## 2 Methods

Evaluation of the M&E/A framework was part of the overall evaluation of the GVAP, the methods of which are described in an accompanying article in this issue of the journal [4]. The evaluation consisted of semi-structured interviews with 80 stakeholders and surveys to which over 300 individuals responded. The interviews and surveys targeted immunization stakeholders at the global, regional and country levels. This article focuses only on the stakeholder feedback on the M&E/A framework.

## 3 Results

**Fig. 1:**
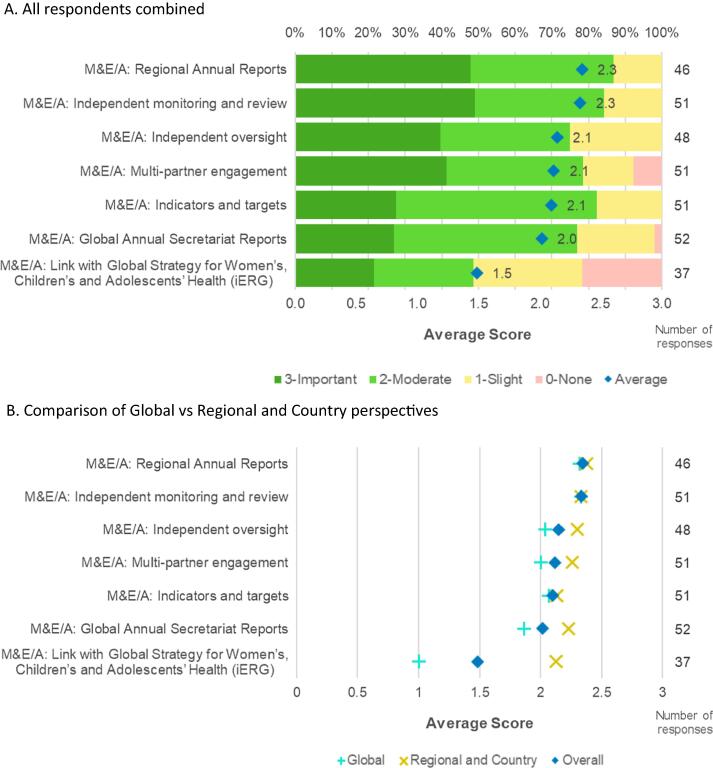
**Summary of feedback from the online stakeholder survey.** Perceived GVAP contribution to improving global immunization: score distribution and average score for each of the survey questions relevant to M&E/A. A. All respondents combined. B. Comparison of Global vs Regional and Country perspectives.

As would be expected in any such process, the responses were mixed and contradictory for some components, especially in the in-depth interviews. The responses through the online survey conducted in 2019 indicated a predominantly positive response to the M&E/A framework, process and outcomes (Fig. 1). In this survey, respondents were asked to rate the contribution of GVAP M&E/A activities to accountability for immunization on a scale of 0 – 3, with 3 representing an important contribution, 2 representing a moderate contribution, 1 representing a slight contribution, and 0 representing no contribution. In contrast, in the interviews, respondents had more opportunities to comment on the limitations of the M&E/A framework, process and outcomes.

### 3.1 Overall framework

The feedback from the interviews indicated that the M&E/A framework and process was a highlight of GVAP. Interview respondents indicated that the M&E/A framework was a step in the right direction and one of them stated that this was the first time there was a common framework for all regions and countries. It was also noted that through the use of the framework, M&E was mainstreamed with most countries contributing data. However, it was also noted that the framework was more adapted for countries that have the resources since the necessary investments from national governments and their partners to implement the recommendations was lacking in many resource-poor countries. Six of seven survey questions relating to M&E/A received scores between 2 and 3, indicating moderate to important contributions to the success of the GVAP, somewhat higher than questions relating to SOs [4].

### 3.2 Indicators and targets

GVAP adopted existing global goals and targets established through resolutions in the WHA or WHO Regional Committee (RC) meetings. However, some of the goals were seen as aspirational and beyond the reach of many countries. Several interview respondents highlighted the need to balance global aspirations with the feasibility for achieving them based on regional and national realities. While a few respondents stated that the GVAP goals should be carried forward, many respondents stated that the goals and targets should be revisited and made more realistic or suggested a bottom-up approach to setting goals and targets.

In general, the indicators were perceived as being well-defined and appropriate. They served as a benchmark and a reminder for every level on what is important and what to focus on to assess progress. However, it was felt that the financing indicators needed to be improved. A few respondents expressed concern that there were too many indicators or that the outputs of some indicators were difficult to interpret and did not lead to any meaningful recommendations.

Reflections on the new indicators and targets in the GVAP, i.e. those that were not carried over from existing goals and targets previous immunization strategies or established through WHA or RC resolutions, were also positive. The Research & Development (R&D) indicators were perceived as being based on a more realistic assessment of R&D processes and timelines. The new indicators such as those measuring vaccine stockouts and confidence and demand for vaccination were perceived to have drawn attention to these issues and highlighted their role in vaccination coverage.

Some respondents also indicated that the indicators in general did not provide enough information on the root causes to allow more targeted recommendations for corrective actions.

### 3.3 The impact of the M&E/A process and reports

One of the important contributions of the M&E/A reports was the focus it brought on the issue of data quality, starting from the very first report.[5] The reports highlighted the importance of data quality to effectively monitor progress while also acknowledging the challenges with the collection, reporting, analysis and interpretation of data. The reports also served to bring greater attention to the quality and use of subnational data, with one respondent noting that there was also an important shift from a focus on geography alone to also considering the attributes of communities, e.g. ethnic composition or socio-economic status.

While several respondents spoke positively about the SAGE recommendations for corrective actions and about implementing them or using them for advocacy purposes, others felt that the recommendations were not specific enough to be actionable and that there were no mechanisms or resources to follow up and monitor the implementation of the recommendations at the country level.

There were mixed responses on the visibility of global and regional assessment reports, with a few country level respondents claiming that they were unaware of the reports and that they were not visible at the country level. Others, at global, regional and country levels, showed awareness of the reports and were able to cite examples from them. It was reported that regional immunization meetings were sometimes organized around the reports, with pressure being applied on countries who were falling short of targets set by the respective RITAGs. Even when there was awareness of the reports, there was a perception that they were not fully read. However, the interventions during the WHA indicated that at least a few people in the country had read the SAGE assessment reports in detail. The responses from the survey also indicated the high impact of the global and regional assessment reports (Fig. 1).

There were mixed responses on the annual reporting process through the WHA with some respondents indicating that the annual WHA discussions served to keep immunization high on the agenda and focused the attention of the Ministers on the key issues. Some respondents felt that the discussions at the WHA and its side meetings served to create peer pressure and draw attention to countries that were not making progress. Others were more sceptical and felt that once the Ministers returned to their countries, there was no follow up action. It was pointed out that depending on the WHA as the “sole touch point” for communications was too narrow an approach and might reinforce existing dynamics, while what was needed is a shift towards greater country ownership.

The feedback from the interviews highlighted the failure of the accountability process, though it was unclear what the expectations were in terms of holding stakeholders accountable, especially at the global level. At least some responses in the interviews demonstrated a lack of clarity as to who is to be held accountable for such a broad immunization agenda. Several respondents compared GVAP accountability with the Global Polio Eradication Initiative accountability process, which was considered to be more successful. One respondent clearly felt that unless there was a “financing whip”, accountability would be difficult to implement. There were suggestions that there should be a shift to greater country ownership in the monitoring and review processes to improve accountability. There were also suggestions that all stakeholders, including non-Governmental stakeholders, should be held accountable. One suggestion was to engage with regional institutions such as the African Union, national parliaments and CSOs to create political will and put pressure on national governments to improve governance and accountability.

In contrast to the feedback from the interviews, the survey results reflected a more positive view and suggested that the regional and global monitoring and review processes and the discussions at the Regional Committees and WHA did contribute to accountability (Fig. 1a and b).

## 4 Discussion

This evaluation was aimed at gathering stakeholder views and opinions on the GVAP M&E/A framework to inform the development of global immunization agenda for the next decade, the Immunization Agenda 2030 (IA2030). The evaluation did not attempt to gather empiric evidence of the impact of the framework at the country level. The responses from the interviews and surveys were mixed and often contradictory. Though mixed and contradictory feedback is expected in such exercises, and indeed valued, in some instances the responses indicated a lack of awareness of the details of the framework and of how the process was meant to work. This indicates a failure of communications and advocacy about the framework and in managing expectations about what it could and could not achieve.

### 4.1 Were the goals, indicators and targets appropriate?

The feedback from many stakeholder interviews indicated that the goals should be realistic and achievable and that deeper engagement with countries in setting goals was essential for country ownership and accountability. This may necessitate the use of a more evidence-based approach to set timelines and milestones to achieve the right balance between ambition and realism, and a shift away from setting purely aspirational goals.

There were contradictory views on the number of indicators, ranging from having fewer indicators to a call for more indicators to monitor incremental progress. It is to be noted, however, that several of the SO indicators monitored incremental progress, without setting specific targets. In addition, the secretariat reports described the incremental progress even when targets were not met [6]. While some of the indicators provided an indication of root causes, they were not in themselves enough to get a full understanding of the issues underlying successes and failures in achieving goals. An attempt was made to better understand the root causes in selected countries through desk reviews of programme evaluation reports. More detailed country level programme evaluations will be required to understand the root causes and to better inform corrective actions when the monitoring indicators reveal a problem. The WHO guide for conducting an Expanded Programme on Immunization review [7] and the Tailoring Immunization Programme approach [8] provide guidance on conducting more detailed evaluations to identify problems that need corrective actions.

### 4.2 Did the M&E/A framework achieve its objectives?

The feedback indicated that while the M&E/A framework did not fully meet all expectations, it served to keep immunization high on the global health agenda and stimulated efforts to improve data quality. In 2019, SAGE issued recommendations on measures to improve data quality based on a report on the quality and use of immunization data.[9] However, the M&E/A framework failed to promote greater accountability among stakeholders, countries, their immunization partners and international agencies. Given the difficulties of ensuring accountability, which mainly rests at the country level, through processes limited to the global or regional levels, shifting the ownership of the M&E/A process to the country level merits serious consideration.

Similarly, new mechanisms to monitor stakeholder commitments at all levels may need to be explored to assess whether or not they were being met. The possibility of monitoring commitments and holding relevant non-Governmental stakeholders accountable is greater when done at the national, rather than regional or global levels. In countries where they exist, monitoring, evaluation and accountability could be conducted by independent bodies such as national immunisation technical advisory groups and through engagement with the Interagency Coordination Committees.

## Declaration of Competing Interest

The authors declare that they have no known competing financial interests or personal relationships that could have appeared to influence the work reported in this paper.
